# Executive Functioning in Single-Sided Deafness: A Pediatric Comparison with Temporal Lobe Epilepsy

**DOI:** 10.3390/jcm15103978

**Published:** 2026-05-21

**Authors:** Jessica C. Luedke, David Faller, Dana Martino, Kerri Bolivar, Amanda M. Griffin, Peter Isquith, Alyssa Ailion, Rachel Landsman

**Affiliations:** 1 Department of Psychiatry, Boston Children’s Hospital, Boston, MA 02115, USAdana.martino@childrens.harvard.edu (D.M.); alyssa.ailion@childrens.harvard.edu (A.A.); 2 Department of Otolaryngology and Communication Enhancement, Boston Children’s Hospital, Boston, MA 02115, USA; david.faller@childrens.harvard.edu (D.F.); kerri.bolivar@childrens.harvard.edu (K.B.); amanda.griffin@childrens.harvard.edu (A.M.G.); peter.isquith@childrens.harvard.edu (P.I.); 3 Department of Otolaryngology Head and Neck Surgery, Harvard Medical School, Boston, MA 02115, USA; 4 Department of Psychiatry, Harvard Medical School, Boston, MA 02115, USA

**Keywords:** single-sided deafness, unilateral hearing loss, executive functioning, temporal lobe epilepsy, pediatric

## Abstract

**Background/Objectives**: Children with single-sided deafness (SSD) have normal hearing in one ear and are deaf in the other. Navigating complex auditory environments with SSD may cause reallocation of cognitive resources necessary for executive functioning (EF), adding potential cognitive burden to listening, though this is not well understood. To characterize EF in children with SSD, we compared their test performance and everyday functioning on performance-based and caregiver-rated EF measures to normative values and to a group of children with temporal lobe epilepsy (TLE). **Methods**: A retrospective review compared children with unaided SSD (*n* = 45) to a clinically referred TLE group (*n* = 39), all aged 6–16 years old, on performance-based measures including verbal fluency (letter, category), digit span, coding, and the BRIEF general executive composite. In the SSD group, those with congenital and acquired onset were compared across the same performance-based measures and BASC-3 executive functioning composite, and BRIEF2 indexes (cognitive, emotional, and behavioral regulation). Within this SSD group, performance-based and caregiver-rated measures were correlated. **Results**: In the SSD group, caregiver-reported EF and test performance were within age expectations. However, SSD participants with congenital onset had poorer caregiver-reported everyday EF. Children with SSD and elevated caregiver-reported EF had greater challenges on performance measures of auditory working memory. EF profiles were similar in the SSD and TLE groups, except the TLE group showed significantly worse performance on semantic fluency. **Conclusions**: Caregiver-rated EF measures may serve as an important tool for detecting neuropsychological deficits in children with SSD. SSD children with congenital onset may benefit from closer EF monitoring. There was lower performance on digit span backward tasks that require auditory working memory in children with elevated daily EF. More research is needed to determine what factors, such as hearing technology use, contribute to EF in children with SSD. *The term SSD is used throughout this article as a neutral placeholder with respect to the variation of terms used with this population (e.g., deaf, hard of hearing, hearing loss, hearing differences, etc.). SSD is used to be inclusive of all cultural/medical perspectives and identities.

## 1. Introduction

Single-sided deafness (SSD) describes the most extreme case of unilateral hearing loss (UHL), whereby one ear has hearing thresholds within normal limits, and the other ear has hearing thresholds in the severe-to-profound hearing loss range. Incidence of SSD is estimated to be 2–5 in 1000 school-aged children [1]. Children with unilateral hearing loss have limited access to binaural difference cues (i.e., level and timing differences between the two ears), which results in evident difficulties with spatial hearing skills. When compared to age-matched peers with normal hearing bilaterally, these difficulties include poorer sound source localization [2,3,4,5] and reduced speech understanding in noisy listening environments [5,6,7].

It is becoming more well understood that unilateral hearing loss (UHL), including SSD, may place a child at risk for academic underachievement, cognitive deficits, psychosocial issues, and speech and language delays [8,9,10,11,12,13]. Furthermore, children with UHL experience listening-related fatigue to a significantly higher degree than their peers with normal hearing [14]. This may result in reduced cognitive performance [15], particularly on tasks requiring efficiency and sustained focus. Executive functioning (EF) is most commonly defined as a set of higher-order cognitive processes that regulate attention, thinking, behavior, and emotions to facilitate goal-oriented problem solving [16,17]. Primary or core components of executive function include inhibitory control, working memory, and flexibility [18,19]. These basic functions enable individuals to initiate, plan, organize, and monitor their behavior. Although research on EF in children with SSD remains limited, a pilot study comparing children with severe-to-profound unilateral hearing loss (UHL) with their siblings found that children with UHL showed greater difficulties in tasks related to certain aspects of EF, such as letter span [20]. In addition, some evidence suggests that children with UHL experience challenges in working memory compared with typically hearing peers, particularly among those who do not use hearing technology [21]. Adults with SSD have also reported reduced concentration and motivation and greater fatigue, with difficulties in concentration associated with poorer cognitive performance [22].

The interplay between environmental stressors (e.g., listening fatigue and increased demands in complex auditory environments) and neural re-organization in children with SSD may impact children’s ability to efficiently deploy EF skills. Individuals with SSD experience variable auditory access dependent on the acoustic environment. In quiet listening environments, individuals can access speech with good success. However, in poor acoustic environments with interfering background noise and/or reverberation (e.g., classroom, gymnasium, cafeteria, etc.), listeners with asymmetric hearing profiles are at a disadvantage. For example, in a school setting, children with SSD might have a hard time quickly locating a peer who is answering the teacher’s question or recognizing what is being said to them in a crowded hallway. The assessment of EF in children with SSD is therefore particularly important as EF may present differently across environments. Formal neuropsychological testing is completed in a quiet one-to-one environment, which differs substantially to a child’s typical listening environment, where there is greater risk for missed auditory input due to poor room acoustics. Given that observed functioning in everyday life and performance in a one-on-one testing setting are likely discrepant, obtaining both neuropsychological test performance and caregiver ratings of everyday functioning is important for this population [23,24].

From a neurological standpoint, unilateral auditory deprivation in children with UHL is associated with reorganization of language networks [25], which may further increase demands on cognitive and executive systems. Functional neuroimaging studies show reduced activation in regions implicated in auditory processing, as well as EF, learning, and memory in children with UHL [26,27,28]. There is also evidence for reorganization and cross-modal integration, such that children with SSD show decreased activation within the right hemisphere [29]. In adults with UHL, structural findings include reduced gray matter in areas associated with EF (e.g., dorsolateral prefrontal cortex, anterior cingulate cortex) and auditory processing (e.g., superior temporal gyrus, Heschl’s gyrus), with greater reductions observed in those with a longer duration of hearing loss [30].

While it may be helpful to compare children with hearing loss to a normative control group, we have found it informative to also compare children with hearing loss to a clinical control group, as it can further clarify whether observed differences are specifically related to hearing loss rather than to broader characteristics shared across clinical populations [31]. For this study, we chose a group of children with temporal lobe epilepsy (TLE) as this group has well known EF difficulties [32,33]. Additionally, given that TLE frequently involves Heschl’s gyrus and the primary auditory cortex, there may be overlap in the disruption of auditory cortical systems and distributed frontotemporal executive networks. Though the auditory profiles of children with SSD and TLE are distinct, children with TLE may also experience difficulties in auditory-related processing [34]. Additionally, children with TLE show impairment across caregiver-reported EF [35]. They have been found to have deficits in auditory attention, working memory, and verbal and nonverbal memory [32,33]. Overall, despite distinct pathophysiology, both SSD and TLE groups show disruption to temporal and auditory networks, suggesting children with TLE may be a good clinical group for comparison with children with SSD to better understand the latter group’s EF.

The following study aimed to (1) evaluate EF profiles of children with SSD across caregiver-reported and performance-based EF, (2) evaluate associations between caregiver-reported and performance-based measures in children with SSD, and (3) compare EF profiles in children with SSD and TLE.

## 2. Materials and Methods

### 2.1. Procedure

All study procedures were reviewed and approved by the Boston Children’s Hospital Institutional Review Board (IRB) [IRB# P00044769 and P00041372] (Exp. 10/2026 and 2/2027). All SSD participants received neuropsychological evaluations as part of routine clinical care through the hospital’s program serving deaf and hard of hearing children or through an ongoing research study. Neuropsychological evaluation data were obtained through the research study’s registry and retrospective chart review of clinical cases. All participants presented for neuropsychological testing between January 2021 and December 2025. Clinical participants with SSD were (1) referred by the Cochlear Implant Team for a cochlear implant candidacy evaluation, (2) referred by internal hospital providers, or (3) self-referred by families. All participants engaged in a neuropsychological evaluation that included administration of neuropsychological measures and caregiver-reported EF and social–emotional–behavioral questionnaires. Clinically referred participants were administered measures appropriate for their age and presenting concern, resulting in variability in available data. However, the same data points were extracted for all research participants.

TLE participants received neuropsychological evaluations through the hospital’s Center for Neuropsychology for phase 1 surgical evaluation or for routine clinical care through internal neurology referrals. Neuropsychological evaluation data were obtained through retrospective chart review from April 2006 to August 2025. Like clinically referred SSD participants, TLE participants were seen clinically and as such, not all participants were administered the same measures and there is variability in test administration due to age and presenting concern. Of note, as the time span for data collection with the TLE group is longer, some measures used both older and newer editions. For example, some participants completed the original Behavior Rating Inventory of Executive Function (BRIEF; [36]), and later participants completed the newer version, the BRIEF2 [37]. The Global Executive Composite (GEC) score from both editions was included in the analyses as the composite scores from each edition are highly correlated. Similarly, both the WISC-IV [38] and newer WISC-V [39] Coding and Digit Span subtests are included in the analyses. These two versions of the same tasks are also highly correlated.

### 2.2. Participants

Participants included met the study criteria for SSD or TLE and were between the ages of 6–16 years old. A diagnosis of SSD was confirmed by a clinical audiologist. The following audiologic criteria were used: (1) four-frequency pure-tone average (4FPTA) ≥ 60 dB HL in the impaired ear, (2) estimated aided speech intelligibility index (SII) < 0.65 in the impaired ear [40] and/or poor suprathreshold word recognition ability in the impaired ear (<60–correct on a developmentally appropriate speech recognition test), (3) 4FPTA < 25 dB HL in the contralateral/better-hearing ear, and (4) unaided SII > 80 in the contralateral/better-hearing ear [41]. All SSD participants were unaided (i.e., they did not use personal hearing technology) at the time of their neuropsychological evaluation. All participants except one did not wear any hearing technology regularly. One participant wore a traditional hearing aid for less than 4 h a day in the 6 months preceding testing and did not wear the device during the evaluation, so they were classified as “unaided.” The onset of hearing loss was coded for SSD participants by a licensed audiologist as congenital (i.e., onset of hearing loss was at birth) or acquired (i.e., onset of hearing loss occurred after birth) based on the results of the newborn hearing screening and initial diagnostic audiological evaluation. 

Diagnostic information for the TLE group was obtained through the medical record. Additionally, laterality of TLE was confirmed by a clinical neuropsychologist via electroencephalography (EEG) and magnetic resonance imaging (MRI) findings at the time of the child’s neuropsychological evaluation. All patients received typical hearing screening through school and/or their pediatrician, and no hearing concerns were reported in their medical records.

All participants endorsed English proficiency and completed all tests in spoken English.

### 2.3. Performance-Based Neuropsychological Measures

#### 2.3.1. Digit Span

The Digit Span subtest from the Wechsler Intelligence Scale for Children, 4th and 5th Editions (WISC-IV, WISC-V [38,39]) assesses auditory attention and working memory. This subtest has three components: Digit Span Forward, Digit Span Backwards, and Digit Span Sequencing. Digit Span Forward is a measure of short-term auditory attention and working memory whereas Digit Span Backwards and Sequencing assess working memory/mental manipulation [39,42]. Digit Span is a verbally mediated measure of EF.

#### 2.3.2. Coding

The Coding subtest from the WISC-IV/V is a measure of processing/graphomotor speed. The Coding subtest requires sustained attention, working memory, and mental flexibility. This task is a measure of primarily visually mediated EF.

#### 2.3.3. Verbal Fluency

The Verbal Fluency (VF) subtest from the Delis–Kaplan Executive Function System Functioning (D-KEFS; [43]) assesses multiple aspects of EF (e.g., initiation, self-monitoring, and mental flexibility). Subcomponents of this test assess Letter Fluency, Category Fluency, Switching, and Switching Accuracy. Only Letter Fluency and Category Fluency are used in the current study. VF is a measure of verbally mediated EF. 

### 2.4. Caregiver Rating Questionnaires

#### 2.4.1. BASC-3 PRS

The Behavior Assessment Scale for Children, 3rd Edition, Parent Rating Scales (BASC-3 PRS; [44]) is a standardized, multidimensional caregiver-report measure of externalizing, internalizing, behavioral symptoms, and adaptive living skills. The BASC-3 PRS has forms for different age ranges including preschool (2 through 5), school-age (6 through 11), and adolescent (12 through 21). For purposes of this study, we used the Executive Functioning Composite on the BASC-3 PRS school-age and adolescent versions, which have high internal consistency (Cronbach’s alpha range of 0.88–0.93). Questions that compose the index score include items that ask about a child’s attention, organization, and impulsivity.

#### 2.4.2. BRIEF/BRIEF2 Parent Form

The Behavior Rating Inventory of Executive Function, 1st and 2nd edition (BRIEF/BRIEF2) Parent Form is a rating scale completed by caregivers about a child that assesses EF and self-regulation in children and adolescents between ages 5 and 18 [36,37]. Composite indexes were evaluated within the SSD group, including the Behavior Regulation Index (BRI), Emotion Regulation Index (ERI), and Cognitive Regulation Index (CRI). For the comparison between the SSD and TLE groups, we examined only the Global Executive Composite (GEC). 

### 2.5. Data Analysis

Prior to data analysis, we evaluated the data for normality and outlier presence. All kurtosis values fell within the range of −0.75 to +0.75 [45], beyond one value (ERI) at −1.11, indicating acceptable levels for normality. The QQ plots visually confirmed normal distribution, and no outliers were identified. All analyses were performed using SPSS Version 29.0.

In the SSD sample, means and standard deviations were computed for all measures. This included mean scaled scores for all performance-based measures of EF that have a mean of 10 and standard deviation of 3, where lower scores reflect worse performance on the measure. T-scores were calculated with a mean of 50 and standard deviation of 10 for caregiver-report questionnaires including the BASC-3 PRS Executive Functioning index and composite indexes of the BRIEF/BRIEF2, where higher scores reflect greater difficulties. Standardized scaled scores and T-scores were derived using normative test data, allowing performance to be interpreted relative to a normative sample. Scaled scores of 7 or lower and T-scores of 60 or higher reflect greater difficulties [36,37,44,46].

SSD participants with congenital onset of hearing loss were compared to those with acquired hearing loss on all measures. Two separate multivariate analyses of variance (MANOVA) were completed, one for performance-based measures and one for caregiver rating scales. Chi-square analyses were then used to determine if there was a difference in the proportion of those that scored at least one standard deviation below the mean (i.e., scaled score of 7 or below, considered below average) on performance-based measures between groups. Additionally, chi-square analyses were used to determine if there was a difference in the proportion of those with elevated scores (i.e., mean T-score of 60 or above) on caregiver-report measures between groups. 

Pearson correlations were conducted to explore the relationship between caregiver-reported and performance-based measures within the whole SSD group.

Participants with SSD were compared to those with TLE on all performance-based measures. The SSD and TLE groups were compared on demographic variables including age (T-test), ethnicity (chi-square), and race (chi-square). Due to differences in BRIEF versions and low number of BASCs in the TLE group, only the GEC was compared between groups. Two separate MANOVAs were completed, one for performance-based measures, and one for the GEC. Chi-square analyses were used to determine if there was a difference in the proportion of those that scored at least one standard deviation below the mean on performance-based measures between groups. Additionally, a chi-square analysis was used to determine if there was a difference in the proportion of those with clinically elevated scores on the GEC between groups. 

## 3. Results

### 3.1. Demographics

This retrospective study included 45 children (18 Female, M_Age_ = 11.2, SD_Age_ = 3.2, Range, 6–16) with a diagnosis of SSD who did not use personal hearing technology. One participant reported inconsistent hearing aid use during school; the hearing aid was not worn on the day of the neuropsychological evaluation, and the participant was therefore classified as an unaided listener, as described above. For the clinical comparison group, data from 39 children with TLE (15 female; M_Age_ = 11.4, SD_Age_ = 2.4, Range, 8–15) seen clinically were included. Demographic information for each group is presented in Table 1. Most SSD and TLE participants reported their race as White (80% and 82%, respectively) and ethnicity as non-Hispanic (89% and 87%, respectively). All participants communicated in spoken English. There were no significant differences between groups by age, ethnicity, or race.

### 3.2. SSD Descriptives

Mean scores for the SSD participants across performance-based and caregiver-rated EF measures are reported in Table 2. Elevated scores were coded as T scores >59 and below average scores were coded as scaled scores <8. Mean scores on all performance-based tasks were within normal limits (i.e., scaled scores between 8 and 11, reflecting the 25th to 75th percentile ranks). Similarly, mean scores on all caregiver-reported tasks were within normal limits (i.e., T-scores below 60).

### 3.3. Hearing Loss Onset and Executive Functioning

There was not a statistically significant difference in performance-based measures between those with congenital versus acquired onset of hearing loss, *F* (1,31) = 1.04, *p* = 0.401, partial η^2^ = 0.119 (Table 3). The proportion of participants with deficits in performance-based tests did not differ by group (*p* > 0.05).

When evaluating caregiver rating scales, the overall MANOVA was not significant, *F* (1,36) = 1.11, *p* = 0.365, partial η^2^ = 0.11 (Table 4). However, there was a significant difference in BRIEF2 ERI between groups, such that the congenital SSD group had higher (poorer) ratings, *F* (1,36) = 4.32, *p* = 0.044, partial η^2^ = 0.10. The proportion of participants with elevated caregiver ratings were significantly different between groups for the BRIEF2 BRI, χ^2^ (1, N = 41) = 3.90, *p* = 0.048, and BRIEF2 CRI, χ^2^ (1, N = 41) = 5.990, *p* = 0.014, such that the SSD Congenital group had higher elevations compared to the SSD Acquired group. This was not observed for the BASC-3 EF scale or BRIEF2 ERI. 

### 3.4. Associations Between Performance-Based and Caregiver-Reported Measures in the SSD Group

Correlations between performance-based and caregiver-reported measures are reported in Table 5. In the SSD group, BRIEF2 Indexes were significantly associated with Digit Span, primarily due to associations with the Backwards condition, such that higher elevations on the BRIEF2 were associated with worse performance on Digit Span Backwards. These associations were not seen in the Forwards condition. Additionally, the BRIEF2 Cognitive Regulation Index (CRI) was significantly associated with Coding, with higher concerns on the CRI related to lower Coding performance. No significant associations were found between Letter and Category fluency with caregiver-reported EF. Importantly, these associations were not observed in the TLE group beyond a significant negative association between Coding and the BRIEF/BRIEF2 Cognitive Regulation Index (i.e., higher elevations on the CRI were associated with worse Coding performance). 

### 3.5. SSD and TLE Comparison Group

The TLE group had a tendency toward lower scores on the Coding subtest, *p =* 0.054, although mean performance on other measures was within age expectations across both groups (Table 6). Overall, there was a statistically significant difference in performance-based measures between the SSD and TLE groups, *F* (1,58) = 2.95, *p* = 0.027, partial η^2^ = 0.169. There was a significant difference between groups on the Category Fluency task *F* (1,58) = 8.63, *p* = 0.005, partial η^2^ = 0.124, such that the TLE group showed worse functioning. Notably, the TLE group showed a higher proportion of deficits across the Coding, Letter Fluency, and Category Fluency, although these differences were not significant (*ps* < 0.05).

On the BRIEF/BRIEF2 GEC, the overall MANOVA comparing the SSD and TLE groups was non-significant, *F* (1,79) = 2.31, *p* = 0.133, partial η^2^ = 0.028 (Table 7). Additionally, the proportion of those with an elevated GEC did not differ between groups, χ^2^ (1, N = 81) = 0.322, *p* = 0.570.

## 4. Discussion

Overall, findings suggest performance-based executive functioning (EF) on neuropsychological tasks may not fully capture the real-world EF challenges experienced by children with SSD. Caregivers were more likely to endorse EF concerns on an EF-specific rating scale compared to formal testing and a broad-band behavior measure. While EF was within age expectations across both caregiver-reported and performance-based measures in the SSD group, congenital onset of hearing loss emerged as a potential risk factor for greater EF difficulties in daily life. By comparison, children with TLE exhibited worse semantic fluency, but largely similar performance across other measures of EF in daily life, suggesting somewhat similar EF profiles. 

### 4.1. Executive Functioning in Children with SSD

Children with SSD showed performance on EF measures within age expectations when measures were administered in a quiet, controlled testing environment. Electroencephalography (EEG) research indicates children with bilateral asymmetric hearing loss experience a higher cognitive load in noisy environments based on higher alpha power levels [47]. Furthermore, a review of hearing loss and EF [48] highlights variability across studies, linking performance differences to environmental demands. In quiet conditions, children with SSD are likely to perform better on performance-based EF measures.

On caregiver-reported measures, children with SSD again showed EF within normal limits. There were significant associations between caregiver-reported EF and performance on graphomotor speed and auditory working memory/mental manipulation, such that children with higher rated difficulties in daily EF showed worse working memory performance and graphomotor speed. Given this association, evaluating EF may be an efficient way to measure a child’s risk for EF difficulties that may warrant further assessment (i.e., a full neuropsychological evaluation). Some children exhibit auditory working memory vulnerabilities, particularly in noisy environments related to the impact of UHL on auditory measures and/or listening fatigue [14,49]. Understanding how to screen with caregiver rating scales before providing all children with SSD neuropsychological testing could be beneficial as caregiver rating scales are often more sensitive to executive concerns [50] and may be more ecologically valid when assessing EF in children with SSD compared to the current performance-based EF measures, which are compared in quiet, one-on-one settings.

### 4.2. Onset of Hearing Loss and Executive Functioning

Children with congenital onset demonstrated greater caregiver-reported difficulties in behavior and cognitive regulation. Although performance-based EF was within age expectations across groups, these findings suggest that children with congenital onset may experience greater EF difficulties in everyday contexts. Importantly, these differences were only observed on a specific EF measure and not a broad-band measure that includes an EF composite (BASC-3), suggesting these difficulties are best captured on a specialized measure of EF in daily life. The duration of hearing loss provides an additional variable that may influence cognitive factors, including EF. It is hypothesized that a longer duration of deafness associated with congenital hearing loss may contribute to this difference. This aligns with research suggesting increased brain-related differences in those with UHL with a longer duration of hearing loss [30]. 

### 4.3. Comparison Between SSD and TLE

Compared to children with SSD, children with TLE showed worse performance on semantic fluency, although the overall proportion of children with a deficit in semantic fluency between groups was not significantly different. Overall, deficits in semantic fluency in the TLE group align with known temporal lobe contributions to language and auditory processing [51,52,53,54,55,56]. There were higher proportions of children with TLE with deficits on phonemic fluency and graphomotor speed, although these differences were not statistically different, and both groups showed similar caregiver-rated EF. As such, the SSD group showed a largely similar EF profile to a clinical group of children with known auditory processing and EF deficits.

### 4.4. Constraints on Generality, Limitations, and Future Directions

To our knowledge, this is the first study to evaluate the profile of EF in children with SSD across caregiver-reported and performance-based measures. However, there are limitations that should be considered when interpreting the results of our study. First, our sample included participants who were primarily white and non-Hispanic, with English as one of their primary languages. As such, results may not generalize fully to the larger SSD population in the United States. Future research in diverse samples, including the impact of bilingualism on the development of EF in children with SSD, is warranted. While our study had a moderate sample size for statistical analysis, it is important to keep in mind that SSD is a low-incidence, rare, and understudied population. Additionally, the SSD population included variability in etiology type (e.g., TBI, nerve deficiency), many of which were unknown. This heterogeneity may have impacted results, as different etiologies could have a differential impact on cognitive functioning and EF. Although onset of hearing loss was evaluated in the current study, evaluating the duration of single sided deafness should be considered as an additional factor that can influence EF. 

Due to the retrospective design, not all participants received all performance-based measures, particularly among participants seen for clinical care who did not participate in the SSD research study, which reduced the sample size across MANOVAs. Different versions of the BRIEF/BRIEF2 and WISC-IV/WISC-V were collapsed for analyses within the TLE group, which creates an additional source of potential bias (although there were no significant differences in scores between versions). Overall, this is a common consideration in clinical research, but future prospective research will be important to understand the EF of children with SSD to confirm our findings.

A more thorough evaluation of performance-based EF within silent vs. noisy environments may clarify the specific difficulties faced by children with SSD within the classroom/home environment, as children with UHL have varying performance on EF tasks dependent on their environment [21]. Additionally, examining associations between performance-based EF in noisy environments as well as teacher- and caregiver-reported EF may clarify whether caregiver-reported measures more effectively capture real-world vulnerabilities in this population. In addition, there is a risk for misdiagnosis of attention-deficit/hyperactivity disorder (ADHD) in children with hearing loss, and this risk has not been explicitly studied in children with SSD. In the general pediatric hearing loss population, there are inattentive and impulsive behaviors that can be misinterpreted when accounting for environment scanning and listening fatigue [57,58,59]. Evaluation of ADHD and EF concerns by providers who understand the impact of hearing loss (e.g., attention being impacted by listening fatigue) is best, but more understanding and dissemination of research regarding the overlap and differences is an important next step for this work in order to educate providers who do not have dual expertise in hearing loss and psychology. 

In an effort to reduce the negative auditory consequences of SSD, cochlear implants (CIs) are being considered more often to minimize aural preference, improve sound localization, improve hearing-in-noise, improve hearing-related quality of life, and reduce listening effort in children with SSD [7,60,61,62,63], although some children with CIs continue to have more difficulties in these areas compared to peers with normal hearing [60,62,63,64,65]. Further investigation into the EF profiles of children with SSD who receive CIs is warranted, especially to consider pre-existing EF vulnerabilities and EF outcomes post SSD CI to ensure appropriate counseling regarding benefit or potential lack thereof on EF post SSD CI.

The present study used a pediatric TLE comparison group due to overlapping challenges in auditory and language processing, despite distinct underlying pathophysiology. Of note, antiseizure medication use in the TLE group may have impacted results, particularly in graphomotor speed [66], and results should be interpreted with this consideration. Future research should consider shared etiological factors, such as congenital cytomegalovirus (CMV), which is associated with both SSD and epilepsy, to better understand how co-occurring neurological risk may impact EF. Additional studies including comparisons with typically hearing peers and children with bilateral hearing loss, who are known to exhibit greater EF difficulties associated with language [67], should be conducted to better contextualize the neurocognitive outcomes associated with SSD. Having a matched typically hearing control group will aid in distinguishing the impact of hearing loss on EF and strengthen the applicability of the current study’s findings. 

## 5. Conclusions

Overall, these results emphasize the importance of collecting caregiver information on an EF-specific measure in children with SSD, as this may be an efficient means of detecting children with SSD who would benefit from an in-person comprehensive neuropsychological evaluation, particularly in children with a congenital onset of hearing loss. Children with SSD may experience EF challenges mostly in adverse listening environments experienced in their home and school settings, and caregiver measures may better capture their EF outside of a quiet, one-to-one environment/in the real world.

## Figures and Tables

**Table 1:** SSD and TLE demographics.

Demographics	SSD (*n* = 45)	TLE (*n* = 39)
Age, M (SD)	11.2 (3.2)	11.4 (2.4)
Sex, N (%)		
Female	18 (40%)	15 (38%)
Male	27 (60%)	24 (62%)
Race, N (%)		
White	36 (80%)	32 (82%)
Other	1 (2%)	2 (5%)
Black	1 (2%)	2 (5%)
Asian	2 (4%)	2 (5%)
Multiple Races	5 (11%)	0 (0%)
Declined/Unknown	0 (0%)	1 (3%)
Ethnicity, N (%)		
Non-Hispanic	40 (89%)	34 (87%)
Hispanic	4 (9%)	1 (3%)
Declined/Unknown	1 (2%)	4 (10%)
Language Use, N (%)		
English	45 (100%)	39 (100%)
Side of HL/TLE, N (%)		
Right	23 (51%)	10 (26%)
Left	22 (49%)	29 (74%)
PTA HL Ear, M(SD)	95.3 (16.4)	-
PTA Contralateral Ear, M(SD)	7.9 (4.6)	-
Onset of HL, N (%)		
Acquired	24 (53%)	-
Congenital	21 (47%)	-
Etiology of HL, N (%)		
Cochlear Nerve Deficiency	6 (13%)	-
EVA	4 (9%)	-
Labyrinthitis	3 (7%)	-
cCMV	2 (4%)	-
Temporal Bone Fracture	2 (4%)	-
Inner Ear Abnormality	2 (4%)	-
NF1	2 (4%)	-
Brain Tumor	1 (2%)	-
Charcot-Marie-Tooth 1A	1 (2%)	-
Cholesteatoma	1 (2%)	-
Cogan’s Syndrome	1 (2%)	-
Labyrinthitis; EVA	1 (2%)	-
Meningitis	1 (2%)	-
TBI	1 (2%)	-
Unknown	17 (38%)	-

**Table 2:** SSD full group descriptive statistics.

Measure	N	Mean	SD	% Elevated or Below Average (N Elevated or Below Average/ N Total)
WISC-V Coding	44	9.09	3.19	25.0% (11/44)
WISC-V Digit Span	44	9.39	3.64	25.0% (11/44)
Digit Span Forward	28	9.43	3.63	25.0% (7/28)
Digit Span Backward	28	10.04	3.23	25.0% (7/28)
Digit Span Sequencing	28	9.29	4.35	32.1% (9/28)
DKEFS Letter Fluency	37	9.89	3.88	24.3% (9/37)
DKEFS Category Fluency	37	11.43	3.35	13.5% (5/37)
BASC-3 EF	42	T 49.67	11.19	19.0% (8/42)
BRIEF2 BRI	45	T 50.36	10.52	22.2% (10/45)
BRIEF2 ERI	45	T 54.82	12.13	40.0% (18/45)
BRIEF2 CRI	44	T 51.91	10.64	20.5% (9/44)
BRIEF2 GEC	44	T 52.91	11.40	31.8% (14/44)

**Table 3:** SSD onset of hearing loss and performance-based executive functioning.

	Wilks’ Lambda				F	*p*	η^2^
Multivariate Test	0.88				1.04	0.401	0.119
	SSD Congenital (N = 16)		SSD Acquired (N = 20)				
Subtest	Mean	SD	Mean	SD	F	*p*	η^2^
WISC-V Coding	8.37	2.50	9.80	3.02	2.30	0.139	0.063
WISC-V Digit Span	9.00	3.14	9.40	3.98	0.11	0.745	0.003
DKEFS Letter Fluency	10.19	4.15	9.45	3.71	0.32	0.577	0.009
DKEFS Category Fluency	11.75	3.84	11.15	3.07	0.27	0.605	0.008
Subtest	SSD Congenital % below average (N below average/N Total)		SSD Acquired % below average (N below average/N Total)		χ^2^	*p*	
			
WISC-V Coding	25.0% (4/16)		20.0% (4/20)		0.13	0.720	
WISC-V Digit Span	31.2% (5/16)		20.0% (5/20)		0.17	0.677	
DKEFS Letter Fluency	18.8% (3/16)		30.0% (6/20)		0.60	0.439	
DKEFS Category Fluency	18.8% (3/16)		10.0% (2/20)		0.57	0.451	

Note. MANOVA comparing SSD Congenital and SSD Acquired groups on performance-based measures, and chi-square analyses comparing the proportion of those with deficits on subtests (scaled scores ≤ 7).

**Table 4:** SSD onset of hearing loss and caregiver-reported executive functioning.

	Wilks’ Lambda				F	*p*	η^2^
Multivariate Test	0.89				1.11	0.365	0.110
	SSD Congenital (N = 18)		SSD Acquired (N = 23)				
Scale	Mean	SD	Mean	SD	F	*p*	η^2^
BASC-3 EF	T 53.61	11.612	T 47.09	10.162	3.67	0.063	0.086
BRIEF2 BRI	T 52.11	10.046	T 47.57	8.436	2.48	0.123	0.060
BRIEF2 ERI *	T 58.72	12.271	T 51.22	10.808	4.32	0.044	0.100
BRIEF2 CRI	T 53.33	10.088	T 49.13	7.858	2.25	0.141	0.055
Scale	SSD Congenital % elevated (N elevated/N Total)		SSD Acquired % elevated (N elevated/N Total)		χ^2^	*p*	
			
BASC-3 EF	22.2% (4/18)		17.4% (4/23)		0.15	0.698	
BRIEF2 BRI *	33.3% (6/18)		8.7% (2/23)		3.90	0.048	
BRIEF2 ERI	55.6% (10/18)		26.1% (6/23)		3.69	0.055	
BRIEF2 CRI *	33.3% (6/18)		4.3% (1/23)		5.99	0.014	

* *p* < 0.05. Note. MANOVA comparing SSD Congenital and SSD Acquired groups on caregiver-report indexes, and chi-square analyses comparing the proportion of those with elevated scales (T-Scores > 59).

**Table 5:** Relationship between caregiver-rated and performance-based measures in SSD.

		BRIEF2 BRI	BRIEF2 ERI	BRIEF2 CRI
WISC-V Coding	*r*	−0.176	−0.113	−0.302 *
	*p*	0.253	0.466	0.049
	*N*	44	44	43
WISC-V Digit Span	*r*	−0.329 *	−0.360 *	−0.380 *
	*p*	0.029	0.016	0.012
	*N*	44	44	43
Digit Span Forward	*r*	−0.336	−0.241	−0.349
	*p*	0.081	0.217	0.069
	*N*	28	28	28
Digit Span Backward	*r*	−0.380 *	−0.640 ***	−0.463 *
	*p*	0.046	<0.001	0.013
	*N*	28	28	28
DKEFS Letter Fluency	*r*	−0.007	0.117	−0.041
	*p*	0.966	0.489	0.811
	*N*	37	37	36
DKEFS Category Fluency	*r*	−0.123	−0.097	−0.227
	*p*	0.467	0.568	0.183
	*N*	37	37	36

* *p* < 0.05. *** *p* < 0.001.

**Table 6:** SSD versus TLE performance-based executive functioning.

	Wilks’ Lambda				F	*p*	η^2^
Multivariate Test *	0.83				2.95	0.027	0.169
	SSD (N = 36)		TLE (N = 27)				
Subtest	Mean	SD	Mean	SD	F	*p*	η^2^
WISC-IV/V Coding	9.17	2.85	7.52	3.81	3.86	0.054	0.060
WISC-IV/V Digit Span	9.22	3.59	8.89	2.69	0.16	0.687	0.003
DKEFS Letter Fluency	9.78	3.87	9.00	3.67	0.70	0.405	0.011
DKEFS Category Fluency **	11.42	3.39	8.81	3.73	8.63	0.005	0.124
Subtest	SSD % below average (N below average/N Total)		TLE % below average (N below average/N Total)		χ^2^	*p*	
			
WISC-IV/V Coding	22.2% (8/36)		44.4% (12/27)		3.52	0.061	
WISC-IV/V Digit Span	27.8% (10/36)		25.9% (7/27)		0.03	0.870	
DKEFS Letter Fluency	25.0% (9/36)		37.0% (10/27)		1.06	0.303	
DKEFS Category Fluency	13.9% (5/36)		29.6% (8/27)		2.33	0.127	

* *p* < 0.05. ** *p* < 0.01. Note. MANOVA comparing SSD and TLE groups on performance-based measures, and chi-square analyses comparing the proportion of those with deficits on subtests (scaled scores ≤ 7).

**Table 7:** SSD versus TLE caregiver-reported executive functioning.

	SSD (N = 44)		TLE (N = 37)				
Scale	Mean	SD	Mean	SD	F	*p*	η^2^
BRIEF/ BRIEF2 GEC	T 52.91	11.395	T 56.95	12.497	2.31	0.133	0.028
Scale	SSD % elevated (N elevated/N Total)		TLE % elevated (N elevated/N Total)		χ^2^	*p*	
			
BRIEF/ BRIEF2 GEC	31.8% (14/44)		37.8% (14/37)		0.322	0.570	

Note. MANOVA comparing SSD and TLE groups on BRIEF/BRIEF2 GEC, and chi-square analysis comparing the proportion of those with an elevated GEC between groups (T-Scores > 59).

## Data Availability

The datasets presented in this article are not readily available because the data are part of an ongoing study that uses clinical data not available to the public.

## Abbreviations

The following abbreviations are used in this manuscript:

BASC-3	Behavior Assessment System for Children—Third Edition
BRI	Behavior Regulation Index
BRIEF	Behavior Rating Inventory of Executive Function
BRIEF2	Behavior Rating Inventory of Executive Function—Second Edition
cCMV	Congenital Cytomegalovirus
CRI	Cognitive Regulation Index
DKEFS	Delis–Kaplin Executive Function System
ERI	Emotional Regulation Index
EVA	Enlarged Vestibular Aqueduct
GEC	Global Executive Composite
HL	Hearing Loss
NF1	Neurofibromatosis Type 1
PTA	Pure-tone Average
SSD	Single-Sided Deafness
TBI	Traumatic Brain Injury
TLE	Temporal Lobe Epilepsy
WISC-IV	Wechsler Intelligence Scale for Children—Fourth Edition
WISC-V	Wechsler Intelligence Scale for Children—Fifth Edition
